# Ascofuranone inhibits lipopolysaccharide–induced inflammatory response via NF-kappaB and AP-1, p-ERK, TNF-α, IL-6 and IL-1β in RAW 264.7 macrophages

**DOI:** 10.1371/journal.pone.0171322

**Published:** 2017-02-16

**Authors:** Jun-Young Park, Tae-Wook Chung, Yun-Jeong Jeong, Choong-Hwan Kwak, Sun-Hyung Ha, Kyung-Min Kwon, Fukushi Abekura, Seung-Hak Cho, Young-Choon Lee, Ki-Tae Ha, Junji Magae, Young-Chae Chang, Cheorl-Ho Kim

**Affiliations:** 1 Molecular and Cellular Glycobiology Unit, Department of Biological Sciences, SungKyunKwan University, Seoburo 2066, Jangan-Gu, Suwon, Gyunggi-Do, Korea; 2 School of Korean Medicine and Healthy Aging Korean Medicine Research Center, Pusan National University, Yangsan City, Gyeongsangnam-Do, Republic of Korea; 3 Research Institute of Biomedical Engineering and Department of Medicine, Catholic University of Daegu School of Medicine, Daegu, Republic of Korea; 4 Division of Enteric Diseases, Center for Infectious Diseases Research, Korea National Institute of Health, Heungdeok-gu, Cheongju, Korea; 5 Faculty of Medicinal Biotechnology, Dong-A University, Busan, Republic of Korea; 6 Magae Bioscience Institute, 49–4 Fujimidai, Tsukuba, Japan; 7 Samsung Advanced Institute for Health Sciences & Technology (SAIHST), Sungkyunkwan University, Seoul, South Korea; University of PECS Medical School, HUNGARY

## Abstract

The natural fungal compound ascofuranone (5-chloro-3-[(2E,6E)-7-[(2S)-5,5-dimethyl-4-oxo-tetrahydrofuran-2-yl]-3-methyl-octa-2,6-dienyl]-2,4-dihydroxy-6-methyl-benzaldehyde, MW 420.93) (AF) isolated from *Ascochyta viciae* has been known to promote cell cycle arrest and inhibit invasion of tumor cells. We have previously studied a structurally similar compound ascochlorin (ASC; MW 404.93) with regard to its anti-inflammatory activity in LPS- stimulated RAW 264.7 macrophages. In order to examine the relationship between the anti-inflammatory activities and the molecular differences between AF and ASC, the activity of AF is herein studied, because ASC has a unique trimethyl oxocyclohexyl structure, while AF has a unique dimethyl-oxo-tetrahydrofuran structure. AF dose-dependently inhibited the production of NO and iNOS and the COX-2 mRNA and protein levels in RAW 264.7 cells. In addition, AF suppressed mRNA expression levels of inflammatory cytokines such as TNF-α, IL-6, and IL-1β, as assessed by RT-PCR. AF (30–50 μg/ml) treatment clearly inhibited the nuclear translocation of NF-κB, AP-1 (p-c-Jun) from the cytosolic space. Phosphorylation of IκB, which functions to maintain the activity of NF-κB, was decreased by AF treatment. Moreover, AF suppressed the binding of NF-κB (p65). Inhibition of IkBa phosphorylation and degradation inhibits nuclear translocation of p65. Immunofluorescence confocal microscopy analysis also revealed that translocation of NF-κB and AP-1 (p-c-Jun) was decreased upon AF treatment. AF specifically decreased the expression level of p-ERK, but not the expression level of p-p38 or p-JNK. Given these results, we suggest that AF suppresses the inflammatory response by targeting p-ERK. This indicates that AF is a negative regulator of LPS-stimulated nuclear translocation of NF-κB and AP-1 (p-c-Jun) in RAW 264.7 macrophages, and specifically it targets p-ERK. Therefore, AF and ASC exert their effects in different ways, most probably because their structural differences allow for specific recognition and inhibition of their target MAPKs. Our results further suggest that AF could be a natural bioactive compound useful for treating inflammation-mediated pathological diseases.

## Introduction

Inflammation is a biological defense mechanism which, when dysregulated, causes human diseases including vascular diseases, tumor initiation, arthritic bone diseases, asthma, atopy, allergic rhinitis, and bronchitis [1,2]. During the inflammatory response, monocyte-differentiated macrophages produce pro-inflammatory cytokines such as interleukin (IL)-1β, IL-6, and tumor necrosis factor-α (TNF-α) as well as low molecular weight mediators such as nitric oxide (NO) and prostaglandins (PGs) [1] via inducible nitric oxide synthase (iNOS) and cyclooxygenase-2 (COX-2) [3]. NO is an L-arginine-liberated radical catalyzed by NOS and is a key inflammatory mediator in activated macrophages. iNOS-catalyzed NO is also a mediator of both acute and chronic inflammatory reactions [3]. Furthermore, oxidized forms of NO have been shown to be carcinogenic [4]. COX-2, an inducible enzyme, catalyzes the transformation of arachidonic acid to PGH_2_, which is a precursor of several inflammatory mediators, such as PGE2 and prostacyclin [3], during inflammation [5]. Many human diseases are caused by the overproduction of inflammatory mediators, and, therefore, inflammatory diseases can be prevented and/or treated by effective inhibition of inflammatory mediators [3].

It is well known that the nuclear factor NF-κB family of transcription factors plays a key role in the expression of inflammatory genes such as iNOS and COX-2 [1–3]. NF-κB is present in the cytosolic area of naive cells in an IκB-bound quiescent form [6]. Such factors are regarded as valuable targets for suppressing inflammatory diseases [7–9]. MAPK signaling pathways such as those mediated by p38, JNK, and ERK are important for NF-κB subunit p65 transactivation or translocation [10]. Therefore, NF-κB nuclear translocation is an active inflammatory response, suggesting the possibility for its manipulation in anti-inflammatory drug development [11].

Ascofuranone (AF) is a natural compound isolated from the fungus *Ascochyta viciae* with a structure of 5-chloro-3-[(2E,6E)-7-[(2S)-5,5-dimethyl-4-oxo-tetrahydrofuran-2-yl]-3-methyl-octa-2,6-dienyl]-2,4-dihydroxy-6-methyl-benzaldehyde and a molecular weight of 420.93. AF was initially isolated as an antibiotic from *Ascochyta viciae* [12] that inhibits *Trypanosoma brucei* oxidase. It has been a lead compound in drugs targeting the African sleeping sickness [13], as it inhibits infections *in vitro* and *in vivo* [12]. It has been known to induce cycle arrest and inhibit the invasion of tumor cells, thereby suppressing tumor growth, and to reduce angiogenesis [14–16] and it is known to be involved in immune system modulation [17,18]. The anti-angiogenic capacity of AF is based on disruption of EGF-induced EGFR activation [19] and PI3K/Akt/mTOR activation, in which PI3K/Akt/mTOR signaling induces HIF-1 expression in MDA-MB-231 cells [20]. In an *in vivo* angiogenesis model of C57BL/6 N mice bearing MDA-MB-231 cells, AF effectivity inhibited tumor-related angiogenesis. AF was shown to inhibit the phosphorylation of several intracellular signaling molecules, such as Akt, mTOR, p70S6K, and 4E-BP1. So far, AF has been regarded as a potential anti-cancer drug [20].

To date, several AF derivatives have been synthetically created in order to pharmacologically investigate their effectiveness in tumor regression and anti-metastasis. One structurally distinct derivative is ascochlorin (ASC). Recently, during studies [6] on the anti-inflammatory activity, our group found that ASC, which has a molecular weight of 404.93 (C_23_H_32_O_4_Cl), and is thus a smaller compound than AF, has anti-inflammatory activity in murine macrophage RAW 264.7 cells. In LPS-inflamed RAW 264.7 cells, ASC effectively inhibited NO and PGE2 synthesis, reduced iNOS, COX-2, IL-1β, and IL-6 expression, and downregulated NF-κB, p-ERK1/2, and p-p38. Interestingly, the only difference between AF and ASC is the position of the methyl group in furan or the cyclohexyl group, as observed on the non-chloride-bound side of the two compounds. ASC has a trimethyl oxocyclohexyl structure, while AF has a unique dimethyl-oxo-tetrahydrofuran structure. During investigation of these two compounds, the structural difference in AF was shown to be associated with a similar but a more effective regulatory activity of the c-Jun transcriptional factor and to have anti-inflammatory effects in RAW 264.7 macrophages. In the present study, the anti-inflammatory activity of AF is studied in RAW 264.7 macrophage cells. In addition, in order to examine the mechanistic relationship between the anti-inflammatory activity and the molecular difference between AF and ASC, intracellular signaling and related phosphorylation changes induced by AF are investigated following treatment with 100 ng/ml LPS. This is the first study to report about the anti-inflammatory activity of AF. This study also elucidates the difference between the 2 compounds, ASC and AF, using LPS- treated murine macrophage RAW 264.7 cells.

## Materials and methods

### Reagents

AF was isolated from a fungal strain of *Ascochyta viciae* and was kindly provided by the co-authors Dr Young-Chae Chang, Dae-gu, South Korea and Dr Junji Magae, Tokyo, Japan. The phytopathogenic fungus *Ascochyta viciae* LIBERT was isolated from the agricultural field and deposited at the Mage Bioinstitute, Tokyo, Japan. AF was isolated as a hypolipidemic substance from the fermented broth of the strain [21]. The basic structure of AF is a prenyl-phenolic compound. LPS (*Escherichia coli* 0111:B4), Hoechst staining solution, Griess reagent, antibodies against β-actin, iNOS, COX-2, p-ERK, p38, p-p38, AP-1, NF-κB, and p65, and 3-(4,5-dimethylthiazol-2-yl)-2,5-diphenyltetrazolium bromide (MTT) were purchased from Sigma–Aldrich (St. Louis, MO, USA). JNK and p-JNK antibodies were obtained from Cell Signaling Technology, Inc. (Beverly, MA, USA). ERK antibody was purchased from Upstate (Atlanta, GA, USA). Lamin B antibody was purchased from ABcam (Cambridge, UK). The EMSA kit was obtained from Promega, Inc. (Madison, WI, USA).

### Cell culture and MTT assay

RAW264.7 murine macrophage cells were obtained from American Type Culture Collection (ATCC, Rockville, MD, USA) and cultured in Dulbecco’s Modified Eagle Medium (DMEM, Rockville, MD, USA) containing 10% fetal bovine serum (FBS), 100 units/ml penicillin, and 100 ng/ml streptomycin. Cells were grown at 37°C in a humidified atmosphere containing 5% CO_2_. To evaluate cell viability, RAW264.7 cells were plated at a concentration of 1 X 10^4^ cells/well in a 96-well plate, and they were treated with 0, 10, 30, and 50 μM of AF.

### Effect on Nitric Oxide (NO) production

RAW264.7 cells were seeded in 24-well plates at a density of 1.0 × 10^5^ cells/well. RAW264.7 cells were treated with LPS (100 ng/mL) only and with different concentrations of AF for 24 h; then, the cells were incubated for 24 h at 37°C in a CO_2_ incubator. The effect on NO production was monitored by measuring the nitrite level in the culture medium. This was performed by mixing the medium with Griess reagent. Optical density was determined at 540 nm after 10 min incubation. The nitrite concentration was measured with reference to a standard curve of NANO_2_.

### Reverse Transcription-Polymerase Chain Reaction (RT-PCR)

Total RNAs were extracted using TRIzol reagent (Invitrogen) and subsequently used to generate cDNA using an RT-PCR system (total RNA, 1 μg). Target gene amplification was performed using specific oligonucleotide primers in a normal PCR system. The primer sequences used were as follows:

iNOS, forward (5'-ATGTCCGAAGCAAACATCAC-3') and reverse (5'-TAATGTCCAGGAAGTAGGTG-3'); COX-2, forward (5'-GGAGAGACTATCAAGATAGT-3') and reverse (5'-ATGGTCAGTAGACTTTTACA-3'); IL-6, forward (5'-CCGGAGAGGAGACTTCACAG-3') and reverse (5'-TCCACGATTTCCCAG-AGAAC-3'); IL-1β, forward (5'-TGCAGAGTTCCCCAACTGGTACA-3') and reverse (5'-GTGCTGCCTAATGTCCCCTT-G-3'); TNF-α, forward (5'-TCAGCCTCTTCTCATTCCTG-3') and reverse (5'-TGAAGAGAAC-CTGGGAGTAG-3'); and β-actin, forward (5'-GATCCGTGAAGATCAAGATCATTGCT-3') and reverse (5'-TGATCTTCATTTTTTACGCGTGAATT-3'). PCR products were analyzed on 1.5% agarose gels and bands were visualized using ethidium bromide staining. The band intensity was quantified by scanning with a gel documentation and analysis system (Image J, Bethesda, MD, USA).

### Western blot analysis

RAW264.7 macrophage cells were plated on 24-well plates (5 x 10^5^ cells/well) and treated with or without 1–50 μM AF and 100 ng/mL LPS for 24 h. After incubation, the cells were washed three times in ice-cold PBS (pH 7.4) and whole-cell extracts were isolated in 1% NP-40 lysis buffer containing 1.5 M NaCl, 1 M HEPES (pH 7.45), 100 mM NaF, 100 mM Na-Pyrophosphate, 100 mM Na-Orthovanadate, NP-40 and protease inhibitor cocktail at 4°C on ice for 15 min, and cell debris was discarded by centrifugation. Cytosolic and nuclear proteins were extracted using NE-PERTM nuclear and cytoplasmic extraction reagents (Thermo Scientific) according to the manual. The protein concentration was measured using the Bio-Rad protein assay (Bio-Rad Laboratories Hercules, CA, USA). Proteins (15–40 μg) were separated using a 10% SDS-polyacrylamide gel and were transferred to nitrocellulose (NC) membranes. Each membrane was blocked with 5% skim milk in Tris-buffered saline (150 mM NaCl, 10 mM Tris-HCl, pH 7.5) with 0.01% Tween 20 (TBS-T) buffer. In order to detect the target proteins, the membrane was incubated with primary antibodies specific for iNOS, COX-2, ERK1/2, p-ERK1/2, p38, p-p38, JNK, p-c-Jun, p-JNK, or NF-κB P65 overnight at 4°C. The membrane was then washed with TBS-T buffer and incubated with horseradish peroxidase (HRP)-linked anti-mouse, anti-rabbit, or anti-goat immunoglobulin G secondary antibodies. Detection was `performed using enhanced chemiluminescence (ECL). Detection images were captured using a western imaging system ChemiDOC (Davinch-K, Davinch-Invivo^tm^).

### Electrophoretic mobility shift assay

An electrophoretic mobility shift assay (EMSA) kit (Promega) was used to detect DNA-transcription factor interactions. RAW 264.7 macrophage cells were pre-treated with 50 μM AF for 30 min and stimulated with 100 ng/ mL of LPS for 15 min. After incubation, nuclear proteins were extracted from the cells. Then, 2.5 μg of nuclear protein extract was mixed with the double-stranded NF-κB oligonucleotide (CAGTGGAATTCC CCAGCC) or AP-1 oligonucleotide (CGCTTGATGACTCAGCCGGAA), and T4 kinase-catalyzed end-labelling (TUNEL) was performed in the presence of [γ-^32^P]-ATP. Competition was ensured with a 100-fold excess of an unlabeled oligonucleotide. The binding reaction was carried out for 20 min at 37°C in a hybridization oven. DNA–protein complexes were separated on native 4% polyacrylamide gels that were pre-electrophoresed for 10 min in 0.5× Tris-borate-EDTA (TBE) followed by electrophoresis for 15 min at 300 mV. The gels were vacuum-dried and exposed to X-ray film overnight at -70°C in a freezer.

### Immunofluorescence

RAW 264.7 macrophages were cultured at a sub-confluent density on 12 mm-diameter sterile coverslips in 24 well cell culture plates, pre-treated with 50 μM AF for 30 min, and then treated with or without LPS for 15 min. Cells were fixed with 4% paraformaldehyde in PBS for 20 min, washed three times with PBS, and then permeabilized with 0.2% Triton X-100 in PBS for 20 min at room temperature. Non-specific binding was blocked with 1% bovine serum albumin in PBS for 1 h at room temperature. To investigate the cellular localization of NF-κB and AP-1, NF-κB- and AP-1-specific antibodies (1:500 in 1% BSA in PBS) were added and incubated overnight at 4°C. The cells were washed with 0.1% Tween-20 in PBS and then further incubated with a fluorescein isothiocyanate (FITC)-conjugated anti-rabbit IgG antibody for 3 h at 4°C. For nuclear staining, Hoechst solution was added at a final concentration of 0.5 μg/mL for 10 min in the dark. After a final wash with PBS-T, slides were mounted with anti-fade reagent (Molecular Probes). Images were captured using a fluorescence microscope (LSM 700, AxioObserver, C-Apochromat 63x/1.20 W Korr M27).

### Statistical analysis

All experiment results are representative of at least three independent experiments performed in triplicate. The results of the data were analyzed by one-way analysis of variance (ANOVA), followed by following by post hoc Bonferoni test; p-values of <0.05 were considered statistically significant. *p < 0.05 and **p < 0.01 indicate significant differences from the LPS alone-treated cells.

## Results

### Chemical structure and effects of AF on cell viability in RAW 264.7 macrophage cells

**Fig 1:**
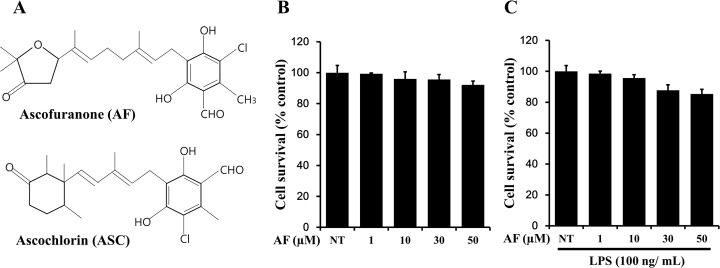
Chemical structure and effects of AF on cell viability in RAW 264.7 macrophage cells. **(**A) Chemical structure of AF. (B), (C) Cells (1 × 10^4^ cell/well) were treated with the indicated concentrations of AF in the absence or presence of LPS for 24 h, and cellular viability was measured by the MTT assay. The data shown are representative of three independent experiments and indicate the mean ± SEM. NT indicates “no treatment”.

A natural fungal compound, ascofuranone (AF) (5-chloro-3-[(2E,6E)-7-[(2S)-5,5-dimethyl-4-oxo-tetrahydrofuran-2-yl]-3-methyl-octa-2,6-dienyl]-2,4-dihydroxy-6-methyl-benzaldehyde, molecular weight, 420.93) was previously isolated from *Ascochyta viciae* and its structure was determined (Fig 1A). The compound is known to promote cell cycle arrest and inhibit invasion of tumor cells, thereby suppressing tumor growth. To date, several AF derivatives have been synthetically created in order to pharmacologically test their capacities for tumor regression and anti-metastasis; among them, AF, a structurally distinct derivative, has been isolated. The structural difference between AF and ASC is observed in the non-chloride-bound side of the two compounds. ASC has a trimethyl oxocyclohexyl structure, whereas AF has a dimethyl-oxo-tetrahydrofuran structure. During the previous investigation of these compounds, AF exhibited a very similar but more effective regulatory activity of the c-Jun transcription factor and had anti-inflammatory effects on RAW 264.7 macrophages. In the present study, in order to examine the cytotoxicity of AF on RAW 264.7 macrophages cells, cells were treated with various concentrations of AF and cell survival was determined using the MTT assay. As shown in Fig 1B, cell death was not observed by treatment with up to 50 μM AF. Cell viability was not changed in the presence or absence of 100 ng/ml LPS, when cells were treated with 0–50 μM AF. Therefore, a concentration of 0–50 μM AF was used for all subsequent experiments.

### Effects of AF on LPS-stimulated NO production in RAW 264.7 macrophage cells

**Fig 2:**
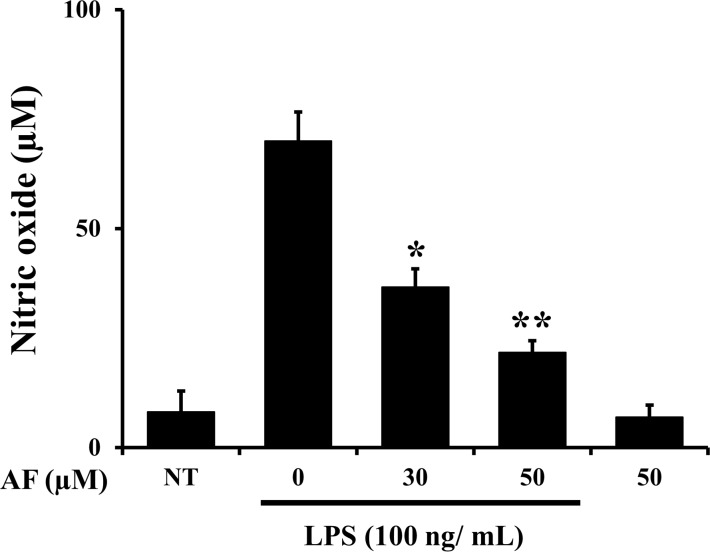
Effects of AF on LPS-stimulated NO production in RAW 264.7 macrophage cells. Cells (5 X 10^5^ cells/well in 24-well pate) were treated with LPS (100 ng/mL) only and with different concentrations of AF for 24 h. The amount of nitrite in the medium was measured using Griess assays. The values are expressed as the means ± SED. of three individual experiments. *p < 0.05 and **p < 0.01 indicate significant differences from the LPS alone-treated cells.

To examine LPS-induced NO production in RAW 264.7 macrophages by AF, cells were treated with LPS (100 ng/mL) only and with different concentrations of AF for 24 h. After 24 h of incubation, the NO levels in the cellular supernatants were assessed using Griess reagent. In RAW 264.7 macrophage cells, LPS activation alone induced iNOS transcription and protein synthesis, and increased NO production. As shown in Fig 2, AF dose-dependently reduced the production of NO in LPS-stimulated RAW 264.7 macrophage cells. Since NO is a signaling molecule that plays a major role in the pathogenesis of inflammation, and is regarded as a pro-inflammatory mediator in abnormal states [3], these results suggest that AF may exert anti-inflammatory effects via reduction of NO in LPS-activated RAW 264.7 macrophage cells.

### AF inhibits the mRNA and protein levels of iNOS and COX-2 in RAW 264.7 macrophage cells

**Fig 3:**
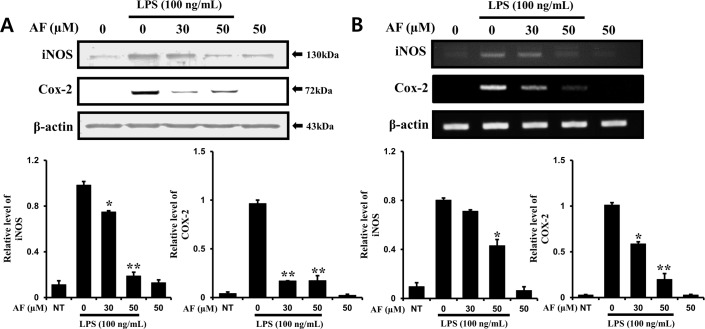
AF inhibits iNOS and COX-2 mRNA and protein levels in RAW 264.7 macrophage cells. RAW264.7 macrophage cells were treated with 0–50 mM AF and then co-treated with LPS for 24 h. Protein (A) and mRNA (B) levels were determined by RT-PCR and Western blot analysis, respectively. The data shown are representative of three independent experiments and indicate the mean ± SEM. *p < 0.05 and **p < 0.01 indicate significant differences from the LPS alone-treated cells.

To evaluate the anti-inflammatory effects of AF, the production of iNOS and COX-2 induced by LPS in RAW 264.7 cells was measured by Western blotting and RT-PCR. After RAW 264.7 cells were cultured for 24 h, the protein and mRNA expression levels of iNOS and COX-2 were measured following treatment with 100 ng/ml LPS and 1–50 μM AF for 24 h. As shown in Fig 3A, iNOS and COX-2 protein expression levels were significantly decreased in a dose-dependent manner in RAW 264.7 macrophage cells treated with AF. Also, the decrease in iNOS and COX-2 mRNA levels was more apparent in cells treated with higher doses of AF (Fig 3B). These results imply that the anti-inflammatory effect of AF is based on the downregulation of iNOS and COX-2 mRNA and protein levels.

### Effects of AF on LPS-induced increases in levels of cytokines TNF-α, IL-6, and IL-1β in RAW 264.7 macrophage cells

**Fig 4:**
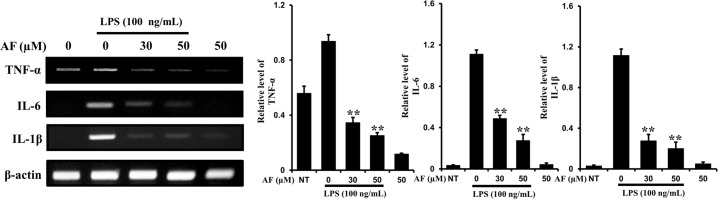
Effects of AF on the levels of LPS-induced cytokines TNF-α, IL-6, and IL-1β in RAW 264.7 macrophages. The mRNA levels of TNF-α, IL-6, and IL-1β were determined by RT-PCR in cells treated with 100 ng/mL of LPS only or with 1–50 μM of AF for 24 h. Band density in Fig 4 (versus β-Actin) is indicated as the mean ± SEM of three independent experiments. **p < 0.01 indicates significant differences from the LPS alone-treated cells.

It is known that during the inflammatory response, murine macrophage cells produce pro-inflammatory cytokines such as TNF-α, IL-1β, and IL-6 [1]. Pro-inflammatory cytokines play pivotal roles in inflammation, including activation of leukocytes and activation of the acute-phase response. As shown in Fig 4, AF suppressed LPS-induced transcription of pro-inflammatory cytokines TNF-α, IL-6, and IL-1β, which are the key inflammatory cytokines.

### Suppressive effects of AF on LPS-stimulated nuclear translocation of NF-κB and AP-1 in RAW 264.7 macrophage cells

**Fig 5:**
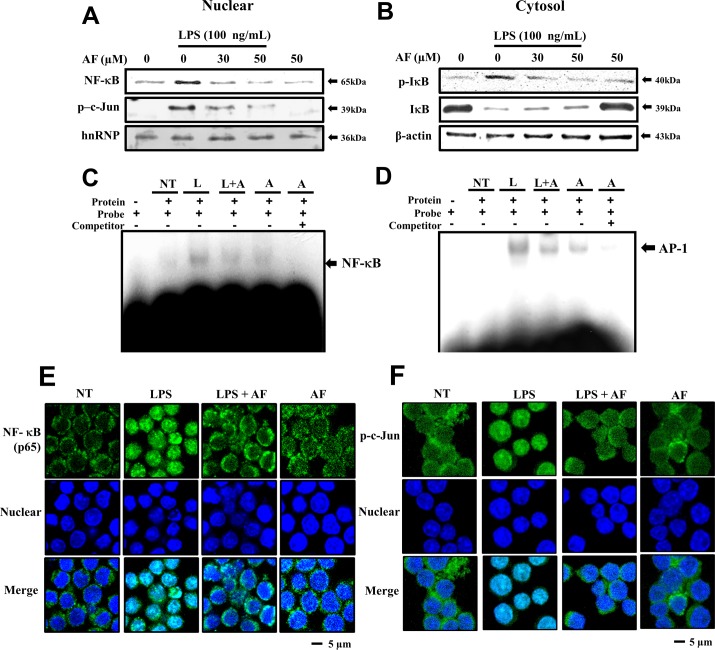
Effects of AF on LPS-induced nuclear translocation of NF-κB and AP-1 in RAW 264.7 macrophage cells. Cells were treated in the presence or absence of various concentrations (0–50 μg/ml) of AF for 30 min and further incubated with or without 100 ng/ml of LPS for 15 min. Then, the cells were harvested and separated into nuclear (A) and cytosolic extracts (B), as described in the Methods section. The protein extracts were separated on SDS-PAGE gels and immunoblotted using Western blot analysis. The antibodies used were anti-NF-κB, -p-c-Jun, -hnRNP, -IκB, and -β-actin. hnRNP and β-actin were used as controls for nuclear- and cytosol-specific proteins, respectively. (C), (D) EMSA showing the reduction in NF-κB and AP-1 DNA binding activity in nuclear proteins. Cells were prepared by stimulation with 100 ng/mL LPS for 15 min and pre-treated with 50 μM AF for 30 min. (E), (F) The translocation of NF-κB (p65) and AP-1 to the nucleus was analyzed by confocal microscopy. Macrophages were immunostained using FITC for NF-κB and AP-1 and Hoechst to label nuclei. White scale bars, 5 μm. NT, no treatment; A, AF; L, LPS; L+A, LPS with AF.

The above results confirmed that AF effectively inhibits LPS-regulated inflammatory responses. Therefore, it was interesting to examine whether AF can also suppress the expression of transcription factors related to inflammatory processes. COX-2, iNOS, and matrix proteinases are known as the important pro-inflammatory proteins, and gene expression of these proteins is controlled by signaling downstream of NF-κB and AP-1 (p-c-Jun) [1–3,22,23]. Therefore, we assessed the ability of AF to inhibit LPS-stimulated nuclear translocation of NF-κB and AP-1 (p-c-Jun) in RAW 264.7 macrophages. As shown in Fig 5A, AF treatment decreased the nuclear translocation of NF-κB and AP-1 (p-c-Jun) from the cytosolic space in a dose-dependent manner. At concentrations of 30–50 μM, the translocation of NF-κB was greatly reduced compared to the basal level in AF-treated cells without LPS. Similar to NF-κB, the translocation of p- c-Jun was inhibited by AF treatment at the same doses. To confirm the reduced level of nuclear translocation, we further investigated the cytosolic presence of IκB, which is a regulator of NF-κB. As expected, the expression level of IκB was increased and the expression level of p-IκB was decreased by AF treatment (Fig 5B). This indicates that AF suppresses LPS-stimulated nuclear translocation of NF-κB and AP-1 (p-c-Jun) in RAW 264.7 macrophages. In addition, as shown in Fig 5C and 5D, AF treatment suppresseds the binding of nuclear transcription factors NF-κB (p65) and AP-1(p-c-Jun) to their consensus DNA cis-elements. These observations suggest that, because AF inhibits the phosphorylation of IκB-α, nuclear translocation of NF-κB p65 was reduced in LPS-activated RAW 264.7 macrophage cells.

In order to observe the reduction in translocation of nuclear transcription factors, we directly analyzed the intracellular behaviors of these factors using a fluorescence microscope. Just as nuclear translocation of NF-κB was inhibited (Fig 5A), immunofluorescence confocal microscopy analysis revealed that nuclear translocation of NF-κB and AP-1 (p-c-Jun) in LPS-treated cells was decreased following AF treatment at the protein level (Fig 5E and 5F). These results indicate that AF is a negative regulator of LPS-stimulated nuclear translocation of NF-κB and AP-1 (p-c-Jun) in RAW 264.7 macrophages.

### AF inhibits ERK1/2 MAPK phosphorylation, but not p38 or JNK phosphorylation, in RAW 264.7 macrophage cells

**Fig 6:**
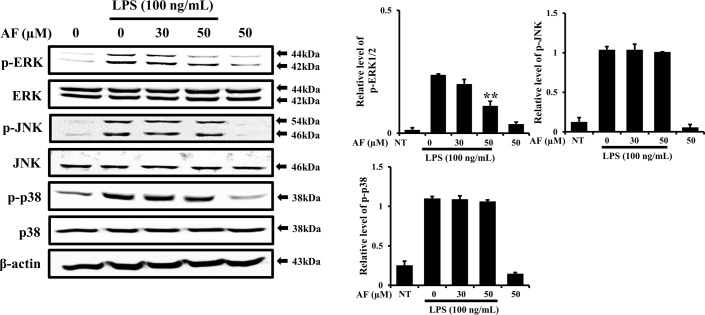
Inhibitory effects of AF on MAPK phosphorylation in RAW 264.7 macrophage cells. Cells were pre-treated with 50 μM AF for 30 min prior to LPS treatment. After treatment with 100 ng/mL LPS for 15 min, the levels of phosphorylated ERK, p38, and JNK were analyzed by immunoblotting. The levels of ERK, JNK, and p38 were estimated by the loaded protein as each control. The data shown are representative of three independent experiments and indicate the mean ± SEM. **p < 0.01 indicates significant differences from the LPS alone-treated cells. Probes: NT, no treatment.

It was previously reported that mitogen-activated protein kinase (MAPK) signaling pathways are related to LPS-activated iNOS and COX-2 expression in macrophages; these are considered the classical pathways that regulate the inflammatory response [24–26]. Activation of MAPK pathways causes releases of inflammatory factors and initiates an oxidative stress response, consequently accelerating the inflammatory process. Therefore, MAPK pathways are regarded as pivotal mechanisms in the regulation of inflammation via production of inflammatory mediators [27]. In order to evaluate whether AF affects MAPK phosphorylation in LPS-treated RAW 264.7 macrophages, cells were pre-treated with AF prior to LPS stimulation and the phosphorylation of MAPKs p38, ERK, and JNK was analyzed As shown in Fig 6, AF showed no effect on the total expression level of p38, ERK, or JNK, but it specifically decreased the expression level of p-ERK in LPS-stimulated RAW264.7 cells; in other words, AF showed a preference for p-ERK, without affecting p-JNK or p-p38. These data show that AF suppresses the inflammatory response through partial regulation of MAPK signaling, by targeting p-ERK, in LPS-activated RAW264.7 macrophage cells. In a previous report on the anti-inflammatory compound ASC, it was suggested that ASC targets both p-p38 and p-ERK [28]. Our present results obtained using AF showed that AF targets only p-ERK. The specificity of AF for p-ERK is interesting when considering the mechanisms of action of these two anti-inflammatory compounds, given their small structural differences. The molecular activity of AF will be our next area of investigation.

## Discussion

Ascofuranone (AF), which is a fungal antibiotic isolated from *Ascochyta viciae*, has a molecular structure of 5-chloro-3-[(2E,6E)-7-[(2S)-5,5-dimethyl-4-oxo-tetrahydrofuran-2-yl]-3-methyl-octa-2,6-dienyl]-2,4-dihydroxy-6-methyl-benzaldehyde and a molecular weight of 420.93 [12]. It exhibits anti-tumor, anti-oxidant, immune-modulatory, and anti-angiogenic activities *in vitro* and *in vivo* [12,14–18]. Regarding angiogenesis, AF was shown to disrupt EGF-induced EGFR activation [19], PI3K/Akt/mTOR activation, and HIF-1 expression in MDA-MB-231 cells [20]. AF also inhibited *in vivo* tumor angiogenesis in C57BL/6N mice bearing MDA-MB-231 cells. Regarding signaling, AF inhibited the phosphorylation of intracellular molecules, including Akt, mTOR, p70S6K, and 4E-BP1, suggesting that AF may be a valuable anti-cancer drug [20]. Because AF is a promising lead compound, researchers have attempted to create synthetic derivatives of AF that can be stably synthesized. The aim is to develop pharmacologically useful compounds that can inhibit tumor growth and metastasis. Among them, ASC is structurally different from AF. The structural difference between AF and ASC is observed in the non-chloride-bound side of the two compounds. ASC has a trimethyl oxocyclohexyl structure, while AF has a dimethyl-oxo-tetrahydrofuran structure.

In a previous report [6], we studied a structurally similar compound ASC (molecular weight, 404.93) (C_23_H_32_O_4_Cl)) in order to investigate its anti-inflammatory activities in RAW 264.7 macrophages. The anti-inflammatory effect of ASC was observed in the early stages of LPS-induced inflammation. It is interesting to observe the difference in pharmacological activity that arises from the difference between the trimethyl oxocyclohexyl group in ASC and the dimethyl-oxo-tetrahydrofuran group in AF. Therefore, in this study, we investigated the anti-inflammatory activity of AF.

The production of NO, a key mediator of inflammation, was inhibited by AF treatment (Fig 2). The prostanoids-producing enzyme COX-2 is directly involved in membrane phospholipidosis triggered by phospholipase A2, and we examined its mRNA and protein levels. AF attenuated the increase in COX-2 protein and mRNA levels in LPS-stimulated RAW 264.7 macrophages in a dose-dependent manner. Furthermore, the mRNA and protein levels of iNOS were also inhibited in LPS- stimulated RAW 264.7 macrophage cells treated with AF in a dose-dependent manner (Fig 3A and 3B). It is known that during the inflammatory response, murine macrophages produce pro-inflammatory cytokines such as IL-1β, IL-6, and TNF-α [29,30]. AF inhibited the expression and production of IL-1β, IL-6, and TNF-α in RAW 264.7 macrophages (Fig 4). Previously, it was reported that a molecularly similar compound ASC also inhibits cytokine expression in the same cells. However, ASC selectively inhibited IL-1β and IL-6, but not TNF-α [6]. It is interesting to observe the differences in the actions of AF and ASC caused by their structural differences. How different chemical groups affect the precise pharmacological activities of these two compounds and lead to differences in alterations of expression of IL-1β, IL-6, and TNF-α in RAW 264.7 macrophages will be studied further. The intracellular pathways involved in inflammatory reactions are complicated, and, thus, the precise mechanism of action by which AF inhibits its cellular targets was investigated. AF suppressed LPS-stimulated nuclear translocation of NF-κB and AP-1 (p-c-Jun) in RAW 264.7 macrophages (Fig 5). In contrast, ASC inhibited the translocation of NF-κB, but not that of AP-1 (p-c-Jun) in RAW 264.7 macrophages [6].

**Fig 7:**
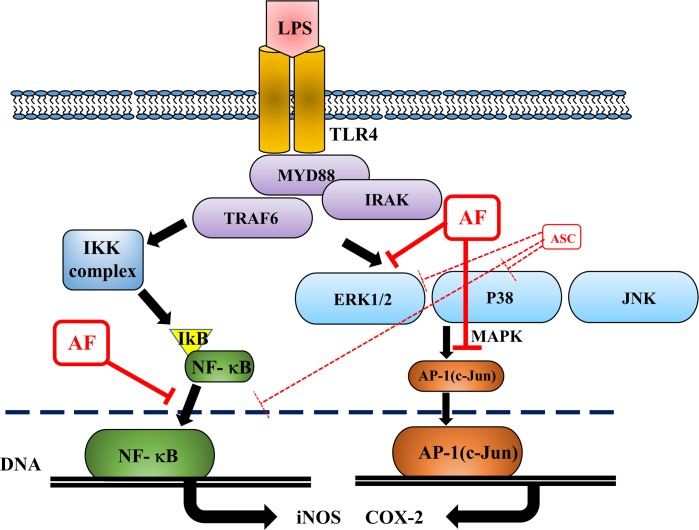
A schematic illustration of the anti-inflammatory activity of AF. AF suppressed the inflammatory response in RAW264.7 macrophages through suppression of NF-κB, AP-1(c-Jun), p-ERK1/2, TNF-a, IL-6, and IL-1B.

MAPK pathways are pivotal in regulation of inflammation and production of inflammatory mediators [31,32]. The present results showed that AF specifically targets p-ERK, which is a very interesting finding. It had no effect on phosphorylation of p38 or JNK. In contrast, ASC effectively suppressed phosphorylation of both p38 and ERK in RAW 264.7 cells [6]. Studies assessing the molecular mechanisms by which the small structural difference between AF and ASC produces differences in specific recognition and inhibition of target MAPKs are underway (Fig 6). In conclusion, as illustrated in Fig 7, we showed that AF suppressed inflammation in RAW264.7 cells via suppression of NF-κB, AP-1(p-c-Jun) activation, and p-ERK1/2. Our results further suggest that AF could be a natural bioactive compound with anti-inflammatory properties that may be a useful agent for treating inflammation-mediated pathological conditions.
